# Maintenance of bone mineral density after implantation of a femoral neck hip prosthesis

**DOI:** 10.1186/1471-2474-9-17

**Published:** 2008-01-31

**Authors:** Ralf Decking, Christoph Rokahr, Matthias Zurstegge, Ulrich Simon, Jens Decking

**Affiliations:** 1 Orthopaedic Department, University of Ulm, Oberer Eselsberg 45, 89075 Ulm, Germany; 2 Orthopaedic Department, St. Francis Hospital, 44155 Muenster, Germany; 3 Asklepios Stadtklinik, Schuetzenstraße 15, 83646 Bad Toelz, Germany; 4 Department of Orthopaedic Surgery, J. Gutenberg University School of Medicine, Langenbeckstrasse 1, 55101 Mainz, Germany

## Abstract

**Background:**

Stress shielding of the proximal femur has been observed in a number of conventional cementless implants used in total hip arthroplasty. Short femoral-neck implants are claiming less interference with the biomechanics of the proximal femur. The goal of this study was to investigate the changes of bone-mineral density in the proximal femur and the clinical outcome after implantation of a short femoral-neck prosthesis.

**Methods:**

We prospectively assessed the clinical outcome and the changes of bone mineral density of the proximal femur up to one year after implantation of a short femoral neck prosthesis in 20 patients with a mean age of 47 years (range 17 to 65). Clinical outcome was assessed using the Harris Hip Score. The WOMAC was used as a patient-relevant outcome-measure. The bone mineral density was determined using dual energy x-ray absorptiometry, performed 10 days, three months and 12 months after surgery.

**Results:**

The Harris Hip Score improved from an average preoperative score of 46 to a postoperative score at 12 months of 89 points, the global WOMAC index from 5,3 preoperatively to 0,8 at 12 months postoperatively. In contrast to conventional implants, the DEXA-scans overall revealed a slight increase of bone mineral density in the proximal femur in the 12 months following the implantation.

**Conclusion:**

The short femoral neck stem lead to a distinct bone reaction. This was significantly different when compared to the changes in bone mineral density reported after implantation of conventional implants.

## Background

The remodelling of the proximal femur after total hip replacement depends on the implants size, geometry and stiffness. Considerable bone resorption in the proximal femur as an answer to stress shielding of the bone surrounding total hip implants has been demonstrated after total hip arthroplasty with a medullar fixation [1-3]. Conventional cementless implants have shown a constant decrease of periprosthetic bone mineral density (BMD) in the proximal femur, as demonstrated by dual-energy x-ray absorptiometry (DEXA) especially over the course of the first year following surgery. Short femoral-neck implants are claiming less interference with the biomechanics of the proximal femur. As such, they may be an alternative to conventional implants, especially for younger patients, where a higher revision rate has been reported. While the mean age of patients requiring a total hip replacement is constantly decreasing, the Swedish Hip Arthroplasty Registry [4] reports an implant survival of 74.9% at 14 years for male patients younger than 50 years, compared to a survival of 84.4% for male patients between the age of 60 and 75 years. Although the reason for failure in the group or young patients is multifactorial, short stemmed femoral shaft prostheses have the theoretical advantage to preserve bone at the initial implantation [5] and ideally maintain this amount of bone over time for upcoming revisions. While long-term results for this group of implants have not been reported, the concept has been supported by biomechanic in vitro research, using composite and cadaver femora models [6-8]. The primary goal of this study was to prospectively investigate the in-vivo changes of bone-mineral density as a parameter of bone remodelling around a short, femoral-neck prosthesis. The secondary goal was to report on its clinical outcome.

## Methods

**Figure 1:**
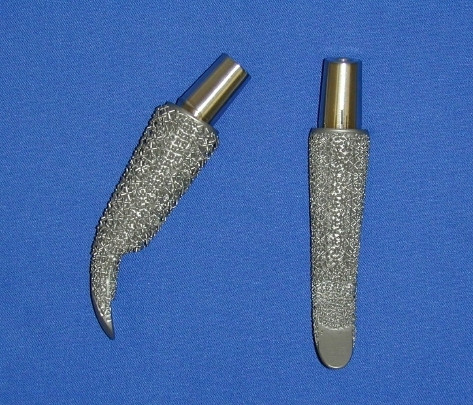
**Cut 2000 femoral neck prosthesis**. The short femoral neck implant used in the study, pictured from the side (left) and from the front (right).

**Figure 2:**
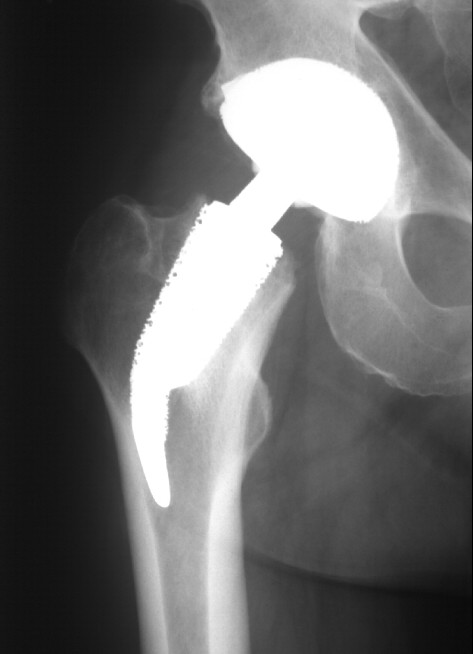
ap radiograph of the Cut prosthesis.

**Figure 3:**
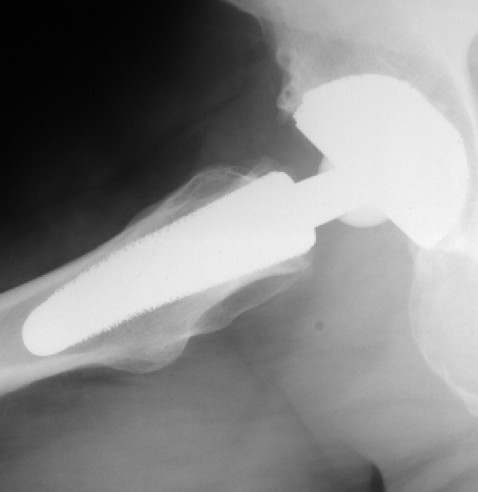
**sagital radiograph of the Cut prosthesis**. Figure 2 is showing the anteroposterior, figure 3 is showing the sagital radiograph 12 months after implantation of the implant in a 50 year old male patient.

Our sample included 20 younger patients who were treated with a cementless total hip replacement. All underwent primary surgery for hip disorders. The femoral implant used in all cases was an ESKA Cut 2000 femoral neck prosthesis (ESKA Implants GmbH & Co, Luebeck, Germany). It is made of CoCrMo alloy and has a macroporous surface structure (Fig. 1). The implant was only indicated for patients at a maximum age of 65 years and a physiological preoperative CCD-angle, where the implant could be placed satisfactory in preoperative templating. For implantation of this "stemless" prosthesis, only the femoral head is resected while the complete femoral neck is preserved to support the implant. Its distal part is meant to firm up on the lateral cortical bone just below the greater trochanter (Fig. 2, Fig 3). A modular conus adapter with adaptable angles and length was used to restore leg length and offset. In all cases, a ceramic head was used in combination with a PE-insert in a cementless press-fit acetabular component. The post-operative treatment regime included weight bearing as tolerated during a 10 to 14-day inpatient stay and a following three-week stay in a rehabilitation facility. At the 3 months follow-up visit, all patients had been able to bear full weight for at least 6 weeks. After institutional review board approval and informed consent, the 20 patients were examined preoperatively, at 10 days, at 3 months and at 12 months after surgery. The Harris Hip Score and the WOMAC were recorded. Both are disease specific tests used frequently in the assessment of total hip replacement [9]. The Harris Hip Score is a classical, staff administered test with domains for pain, function, deformity and motion adding up to a maximum of 100 points. According to Harris[10], following THR a postoperative score of 90–100 points is considered a very good result, while a scores between 80–90 points are good. A score between 70–80 is considered fair, and a score below 70 regarded as bad postoperative outcome. The Western Ontario and McMaster Universities Osteoarthritis Index (WOMAC) is a self-administered questionaire with three subscales measuring pain, stiffness and physical function. A global score may be extracted from the three subscores on a 0 to 10 scale, best to worse. At a three year follow-up of hybrid and cemented total hip replacements, Nilsdotter et a. [11] reported a mean global WOMAC score of 1,8 for the first, and 2,4 for the second group. For this study, the WOMAC's German version was used, which has been shown to be a valid and liable instrument to assess symptoms and physical function disability in patients with hip osteoarthritis [12].

**Figure 4:**
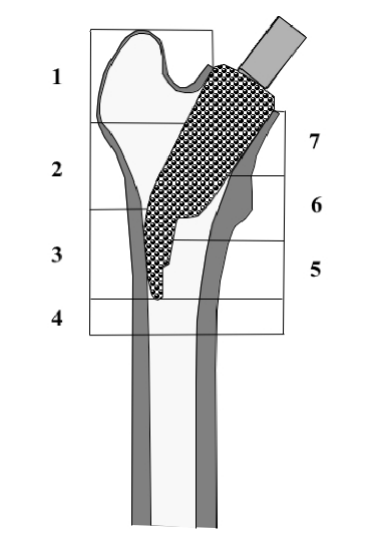
**ROI 1–7 (regions of interest)**. The seven regions of interest for the evaluation of the bone mineral density are shown. For description of the bone mineral density changes on the medial side, ROI 1–3 were combined to one medial value ROI_med_. On the lateral side, ROI 5–7 were combined to one lateral value ROI_lat_. All seven regions of interest were finally combined in one overall value, ROI_all_.

In order to determine the periprosthetic bone density at the hip, DEXA was performed at the 10 day-, 3 month- and 12 month-follow-up, using a Norland Eclipse Scanner (Norland, Ft. Atkinson, WI, USA). Measurements of a calibration phantom were performed daily before scanning of the patients. The scanning procedure as well as the positioning of the patients and the leg were standardized in order to guarantee a high accuracy of the measurements, as requested by Cohen and Rushton [13] and Martini and co-workers[14,15]. Densitometric measurements of the non-operated side were performed on each measurement of the operated hip. These ruled out a possible systemic bone density loss in all of the 20 cases. A software designed for the measurement of bone mineral density adjacent to metal implants was used (Norland DxA Version 3.9.4) on a Norland PC (NPC-200). Seven regions of interest were determined after modification of the classification of Gruen and co-workers to the specific dimensions of the femoral implant [16] (Fig. 4). Because of the relatively small dimensions of the implant, and the congruously small zones compared to conventional prosthesis, the lateral zones 1, 2 and 3 were later combined to a lateral value (ROI_lat_) and the zones 5, 6, and 7 combined to a medial ROI_med_, Bone mineral density around the whole implant was also calculated (ROI_all_). Periprosthetic bone mineral density (BMD) was measured longitudinally at the three postoperative follow-ups. At each measurement, the change in BMD was compared with the baseline 10 days after surgery and calculated as the BMD change in percent in each of the 7 primary and 3 combined regions of interest.

For statistical analysis JMP IN statistical software (SAS Institute Inc, NC, USA) for Macintosh was used in its version 5.1.2. At first the presuppositions for a normal distribution were tested. Since histograms and the Shapiro-Wilk-tests were not able to show a normal distribution in all cases, the Wilcoxon signed-ranks test was used to statistically compare the density changes over the 12 months following surgery. The level of significance was set at alpha = 5%.

## Results

The study included 8 women and 12 men with a mean age at surgery of 47 years (range 17–66 years, standard deviation SD: 11,6 years). There were 12 patients with the implant on the left and 8 patients on the right. The average height of the patients was 174 cm (SD: 10 cm), the average body mass index was 26 (SD: 3.4). There were no radiographic signs of loosening or migration of the femoral or the acetabular components at three months or one year postoperatively. The mean preoperative Harris hip score of 45 points increased to 89 points 12 months after surgery. The global WOMAC score of 5.3 improved to 0.1. Subscores and Global indices of the Harris hip score and the WOMAC are listed in table 1.

**Table 1:** HSS and WOMAC; preoperative and postoperative scores

	**Harris Hip Score preop**		**Harris Hip Score 12 months**	
		SD		SD
**pain**	11.1	7.4	39.6	8.8
**function**	26.9	6.3	43.1	8.6
**deformity**	3.7	0.5	4.0	0.2
**motion**	4.0	0.6	4.9	0.1
**HSS global**	45.6	11.7	88.5	15.1
				
	**WOMAC preop**		**WOMAC 12 months**	
		SD		SD
**pain:**	5.2	1.1	0.8	1.2
**stiffness:**	4.9	1.4	0.9	1.3
**function:**	5.7	1.1	0.8	1.1
**global index**	5.3	1.0	0.8	1.2

Harris Hip Score and WOMAC preoperatively and 12 months after implantation of the stem, n = 20; all values mean; SD: standard deviatiation

### Bone Mineral Density

After surgery the bone mineral density overall slightly decreased in the first 3 months. The highest decrease of little more than 3 % was recorded in the most proximal regions 1 and 7, while the smallest decrease was observed in ROI 3, where the lateral flare of the implant pushes against the lateral cortex. The changes in all regions of interest were statistically significant at 3 months. However, 12 months postoperatively the BMD had leveled off close to the initial values recorded shortly after the index procedure, with the highest increase in ROI 3 laterally (mean +2,8%, SD: 1.4) over the course of one year. All regions on the lateral proximal femur showed a significant change (ROI 1–3, ROI_lat_). The bone mineral density in the different regions and the relative changes in percent are shown in table 2.

**Table 2:** Bone density and changes between measurements at 10 days, 3 months and 12 months postop

**ROI**	**10 days**		**3 months**		**10 d-3 mo**		**12 months**		**10 d- 12 mo**	
	**mean bone density in g/cm2**	***SD***	**mean bone density in g/cm2**	***SD***	**mean change in %**		**mean bone density in g/cm2**	***SD***	**mean change in %**	

**1**	0.76	*0.14*	0.73	*0.13*	-3.35	s	0.75	*0.13*	-0.76	s
**2**	0.81	*0.14*	0.79	*0.14*	-2.99	s	0.82	*0.14*	1.60	s
**3**	1.06	*0.19*	1.05	*0.19*	-1.09	s	1.08	*0.19*	2.84	s
**4**	1.60	*0.23*	1.57	*0.22*	-2.28	s	1.60	*0.23*	-0.35	ns
**5**	1.52	*0.19*	1.49	*0.19*	-3.01	s	1.51	*0.19*	-0.77	ns
**6**	1.51	*0.20*	1.47	*0.20*	-3.71	s	1.50	*0.19*	-0.69	s
**7**	1.08	*0.12*	1.06	*0.12*	-2.77	s	1.08	*0.12*	0.67	ns
**1–3 lat**	0.82	*0.14*	0.80	*0.14*	-2.36	s	0.83	*0.14*	1.37	s
**5–7 med**	1.14	*0.14*	1.10	*0.13*	-3.18	s	1.13	*0.13*	-0.40	ns
**1–7 all**	1.05	*0.14*	1.02	*0.13*	-2.74	s	1.05	*0.13*	0.19	ns

Mean values of bone mineral density and mean values of changes in percent between the 10-day- and 3-month-examinations, as well as between the 10-day- and 12-month-examinations

ROI: Regions of Interest 1–7 and combined zones laterally (1–3 lat), medially (5–7 med) and overall (1–7 all)

SD: standard deviation; s: significant, ns: not significant (Wilcoxon signed-ranks test, t = 0.05)

## Discussion

The present study supports the hypothesis that a short femoral neck stem will lead to a distinct bone reaction, which is fundamentally different to the changes in bone mineral density seen after implantation of conventional implants. While the efficiency of a medullar fixation has been proven during the past decades, it is widely accepted that the BMD decreases especially proximally after total hip replacement using standard designs of the stem. The extent of the changes in BMD seem to correlate on the implants geometry, its size and its stiffness. For example, Yamaguchi and co-workers[17] reported a different pattern of BMD-changes in a proximally-coated and a fully porous-coated stem of otherwise identical design. Both implants used in their study had the same material and surface structure as the Cut, but a different geometry with long stem. Aldinger et al[18] showed a different longitudinal progression in men and women when following the Spotorno stem, which might reflect the reaction to the different stem sizes and stiffness. Alterations of the stem design like the so called anatomically adapted stems which are designed for a proximal force transmission are not able to reduce the proximal bone resorption significantly, as shown by Venesmaa et al. [19]. The same is true for custom made femoral components [2]. All of these studies on conventional stems report on a significant bone density reduction in the proximal part of the femur, regardless of the modifications to the stem design. For better comparison, the percentual changes of bone mineral density in the studies discussed are listed in table 3. Even though a randomized, controlled study with a control-group using conventional stems could not be presented, a comparison to studies on conventional implants support the perception that alternate stem designs are able to reduce or eliminate the stress shielding seen around conventional implants.

**Table 3:** Changes in mean bone mineral density in percent, compared with the first postoperative values

**Author**	**FU listed**	**subgroup**	**ROI 1**	**ROI 2**	**ROI 3**	**ROI 4**	**ROI 5**	**ROI 6**	**ROI 7**
**this study**	12 months	Cut	-0.8	1.6	2.8	-0.4	-0.8	-0.7	0.7
**Yamaguchi et al.**	12 months	fully coated	-18.1	-12.1	-7.8	-8.9	-8.3	-14.5	-21.7
	12 months	proximal coated	-12.3	-8.0	0.3	-3.1	-1.5	-5.3	-17.6
**Aldinger et al.**	12 months	male	-15.5	-10.7	-7.6	-6.9	-6.1	-11.5	-25.0
	12 months	female	-12.0	-2.5	-1.9	-4.7	-3.5	-6.4	-18.8
**Venesmaa et al.**	12 months	anatomic	-10.6	-6.7	-2.0	-4.4	-2.2	-9.3	-22.1
**Leichtle et al.**	6 months	custom stem	-14.9	-13.3	-11.1	-10.7	-10.8	-12.8	-23.7

Note that the ROIs are defined by the implant size. As such, the location of the ROIs for the short femoral neck implant can not be compared directly to the longer implants.

In vitro strain measurements after implantation of the Cut prosthesis have shown less change in the proximal femur when compared to conventional implants. In a experimental setting using strain gauges in cadaver femora [7], strains increased at the strain gauges referring to the ROIs 2, 6 and 7. Since strain gauges only record local strain information on the outer cortical bone surface, they do not fully reflect the in-vivo loading, especially in the cancellous bone of the femur. As such, when comparing the data of the in vivo- and in vitro-results, it is evident that these experimental data have to be regarded with some scepticism. However, even though the distribution of the changes did not perfectly fit the distribution of the BMD-measurements, strain-changes recorded for the short stem in the in vitro study were far smaller than those seen in conventional implants. Steinhauser et al [6] evaluated three different short femoral neck implants in composite femora using photoelastic coating techniques and compared changes in hoop-strains with a conventional implant. In this setting, the strains recorded with the Cut-implant mostly stayed within the 95% confidence intervals of the native composite femora. Nevertheless, there was a clear reduction of stresses medially at the height of the lesser trochanter, where a small, but significant reduction of BMD is seen in the in vitro-data after one year (ROI 6). They also recorded an increase in strains laterally where the lateral tip of the implant pushes against the cortical bone, corresponding to the significant increase in ROI 3 in the in vivo BMD-changes. Munting and Verhelpen showed a similar pattern of strain distribution in a (quite different) experimental stemless prosthesis [8], which used varying trans-trochanteric screw-fixations on the lateral cortex. When a successor of this implant was followed in vivo, the authors also reported on a maintenance of BMD [20] in the proximal femur over the course of up to 6 postoperative years. And Joshi and co-workers [21] used a FEA-model to predict the stresses around a short implant with cables attachments around the greater trochanter. This was able to reduce the stress peaks at the lateral side of the femur. However, the authors concluded, that in vivo and in vitro testing of the prosthesis were still pending.

As a number of clinical studies suggest, the largest part of bone remodelling following total hip arthroplasty ceases within the first postoperative year [20,22,23]. As such, the follow-up in this study should be long enough to show the specific reaction to the short stem observed. Nevertheless, it cannot be ruled out that the changes around this rather untypical implant might have a different course over time. We used a longitudinal study design, as only prospective analysis with the baseline taken after the surgery can provide reliable information about the actual loss of bone density [14,22]. The methods used in our follow-up were standardized and the rotation of the leg was strictly controlled as suggested by studies on the precision of measurement of periprosthetic bone mineral density [24,25].

Conservation of bone stock is an essentially important principle especially in young patients, where the chances for revisions during the patient's lifetime are high. As the data presented here show, an alternative prosthesis design is able to reduce stress-shielding-related bone resorption in the proximal femur. Nevertheless, the authors are aware that other factors influence survival and the clinical results of total joint implants. When Ender and co-workers [26] followed the Cut-stem clinically, they reported on an unacceptable high rate of revisions after a midterm follow- up averaging 5.1 years. This might be due to a difference in the indications for choosing the implant. For our study, the patients were carefully selected, and only young patients with an anatomy believed to be suitable for this special implant were chosen. The implant was not used in patients with coxa vara or valga, nor with an increased anteversion of the femoral neck. All procedures were carefully planned, with the templated stems accurately fitted within the femoral neck and the distal lateral part of the prosthesis firmly against the subtrochanteric cortical bone, and this position was than achieved at the operation. Radiographic and clinical results after the short follow-up of 12 months showed no signs of early failure in this small patient group, and the postoperative HSS and WOMAC-scores match the results seen the follow-up of conventional stems. Other groups using small-sized implants with an intertrochanteric fixation like the Mayo Conservative Hip stem (Zimmer, Inc., Warsaw, IN, USA) reported superior functional results in short-term follow-up when compared to a standard cementless stem [27], as well as an excellent survival of 98% at 10 years [28].

However, the data presented here mainly focus on the bone mineral density changes of a specific stem in a selected group of patients, and does not include any information on the long-term survival of the implant.

## Conclusion

The implantation of a short femoral neck stem lead to a distinctive bone reaction, which differed to the changes seen after implantation of conventional implants. Only further analysis with a longer follow-up of larger patient collectives will be able to show if this is leading to an acceptable survival as well as proven advantages in the case of revision, and subsequently to a clearer view which patients might profit from the use of alternative stem-designs.

## Competing interests

The author(s) declare that they have no competing interests.

## Authors' contributions

RD: study idea, drafting and writing of manuscript, statistical analysis

CR: acquisition of data, analysis and interpretation of data

MZ: acquisition of data, analysis and interpretation of data

US: acquisition of data, analysis and interpretation of data

JD: conception and study design, statistical analysis, figures

All authors have equally contributed to the study, revised the paper for important intellectual contend and have given final approval of the manuscript now submitted.
